# Expansive arterial remodeling of the carotid arteries and its effect on atherosclerotic plaque composition and vulnerability: an in-vivo black-blood 3T CMR study in symptomatic stroke patients

**DOI:** 10.1186/s12968-016-0229-2

**Published:** 2016-03-03

**Authors:** Tobias Saam, Maximilian Habs, Martin Buchholz, Andreas Schindler, Anna Bayer-Karpinska, Clemens C. Cyran, Chun Yuan, Maximilian Reiser, Andreas Helck

**Affiliations:** Institute of Clinical Radiology, Ludwig-Maximilians-University Hospital, Marchioninistr.15, 81377 Munich, Germany; Department of Neurology, Ludwig-Maximilians-University Hospital, Munich, Germany; Institute for Stroke and Dementia Research, Ludwig-Maximilians-University Hospital, Munich, Germany; Department of Radiology, University of Washington, Seattle, WA USA

**Keywords:** Plaque, Remodeling, Stroke, CMR, MRI

## Abstract

**Background:**

Based on intravascular ultrasound of the coronary arteries expansive arterial remodeling is supposed to be a feature of the vulnerable atheroslerotic plaque. However, till now little is known regarding the clinical impact of expansive remodeling of carotid lesions. Therefore, we sought to evaluate the correlation of expansive arterial remodeling of the carotid arteries with atherosclerotic plaque composition and vulnerability using in-vivo Cardiovascular Magnetic Resonance (CMR).

**Methods:**

One hundred eleven symptomatic patients (74 male/71.8 ± 10.3y) with acute unilateral ischemic stroke and carotid plaques of at least 2 mm thickness were included. All patients received a dedicated multi-sequence black-blood carotid CMR (3Tesla) of the proximal internal carotid arteries (ICA). Measurements of lumen, wall, outer wall, hemorrhage, calcification and necrotic core were determined. Each vessel-segment was classified according to American Heart Association (AHA) criteria for vulnerable plaque. A modified remodeling index (mRI) was established by dividing the average outer vessel area of the ICA segments by the lumen area measured on TOF images in a not affected reference segment at the distal ipsilateral ICA. Correlations of mRI and clinical symptoms as well as plaque morphology/vessel dimensions were evaluated.

**Results:**

Seventy-eight percent (157/202) of all internal carotid arteries showed atherosclerotic disease with AHA Lesion-Type (LT) III or higher. The mRI of the ICA was significantly different in normal artery segments (AHA LT I; mRI 1.9) compared to atherosclerotic segments (AHA LT III-VII; mRI 2.5; *p* < 0.0001). Between AHA LT III-VII there was no significant difference of mRI. Significant correlations (*p* < 0.05) of the mRI with lumen-area (LA), wall-area (WA), vessel-area (VA) and wall-thickness (WT), necrotic-core area (NC), and ulcer-area were observed. With respect to clinical presentation (symptomatic/asymptomatic side) and luminal narrowing (stenotic/non-stenotic) no relevant correlations or significant differences regarding the mRI were found.

**Conclusion:**

Expansive arterial remodeling exists in the ICA. However, no significant association between expansive arterial remodeling, stroke symptoms, complicated AHA VI plaque, and luminal stenosis could be established. Hence, results of our study suggest that expansive arterial remodeling is not a very practical marker for plaque vulnerability in the carotid arteries.

## Background

Arterial remodeling has become an important concept for understanding atherosclerosis. Histopathological and imaging studies of coronary arteries showed that it is reasonable to distinguish between inward remodeling, resulting in vessel size reduction with luminal stenosis and expansive remodeling, resulting in an increased vessel area. Expansive remodeling was initially described as a compensatory mechanism that helps to reduce luminal narrowing due to plaque formation in the coronary arteries [1]. However, histopathological specimens and imaging studies in the coronary arteries subsequently have shown that positive remodeling is characteristic for unstable lesions, which are more likely to cause ischemic symptoms [2–5]. In contrast to the endovascular ultrasound-based studies of the coronary arteries, most of the studies on carotid arteries in the past were based either on in-vitro animal models or on autopsy specimen [6–9]. However, these studies do not provide sufficient information about remodeling patterns. On the one hand measurement of dimensions of histopathological is problematic due to the lack of blood pressure in vitro, on the other hand there is the risk of shrinking of the specimens during histopathological processing. Thus, only relatively few and small studies evaluated arterial remodeling of the carotid arteries in-vivo and until now little is known regarding the clinical significance of remodeling in this particular vascular bed. Since it has been shown in various studies that black-blood CMR is capable of precisely evaluating plaque-burden and vessel-diameters [10–12], in this study we sought to evaluate the association between expansive carotid remodeling and ischemic stroke as well as plaque features (AHA-lesion-type, luminal stenosis) using in-vivo black-blood CMR.

## Methods

### Patient selection

Between 2009 and 2011, we consecutively included 111 symptomatic stroke patients in this prospective monocentric observational study. The patients were referred from the Department of Neurology of the Ludwig-Maximilian-University Munich for diagnostic and/or experimental MR imaging of the carotid arteries by black-blood sequences at 3Tesla. Inclusion criteria were defined as: 1) clinical evidence (neurological deficit >24 h duration) and radiographic evidence of acute ischemic stroke on DWI sequences 2) presence of plaques with ≥ 2 mm thickness on one of the carotid arteries as defined by ultrasound 3) written informed consent 4) no contraindications for MR-imaging. The study was approved by the local institutional ethics committee and complied with the declaration of Helsinki. Informed consent has been obtained from each patient.

### Stroke diagnosis and presence of significant luminal stenosis

All patients were diagnosed and classified by the Department of Neurology of the Ludwig-Maximilian-University Munich. The imaging diagnostic work-up included duplex-sonography of the extracranial vessels, transcranial duplex-sonography, and cerebral MRI with diffusion-weighted imaging sequences. Luminal stenosis of the ICA was determined by extracranial duplex-sonography according to the NASCET-criteria (presence of significant luminal stenosis when lumen narrowing >50 %).

### MR imaging protocol

All stroke patients received a multi-sequence black-blood CMR of the carotid arteries (3Tesla, Magnetom Verio, Siemens Healthcare, Erlangen, Germany). The scans were centered on the carotid bifurcation and included TOF-MR-angiography [TR = 21 ms, TE = 3.96 ms], axial pre- and post-contrast, fat- and blood-suppressed 2D-T1 Turbo-Spin-Echo (TSE) sequence [TR = 800 ms, TE = 12 ms], proton-density-weighted [TR = 3000 ms, TE = 13 ms] and 2D-T2 TSE sequence [TR = 3000 ms, TE = 65 ms] sequences (best in-plane resolution 0.5×0.5 mm^2^, slice thickness 1 mm for TOF-images and 2 mm for all other sequences). A dedicated 4-channel surface coil (Machnet B.V., Eelde, Netherlands) was used. Parallel imaging was used for all sequences with a PAT factor of 2 resulting in a total scan time of 15–25 min per patients. For contrast enhanced T1-images, intravenous Gadolinium-DTPA-BMA (Gadobutrol, Bayer-Schering, Leverkusen, Germany) of 0.1 mmol/kg was administered at a rate of 3 ml/s. Post-contrast T-1W imaging was performed approximately 5 min after intravenous injection of the contrast agent.

### MR imaging review

**Fig. 1:**
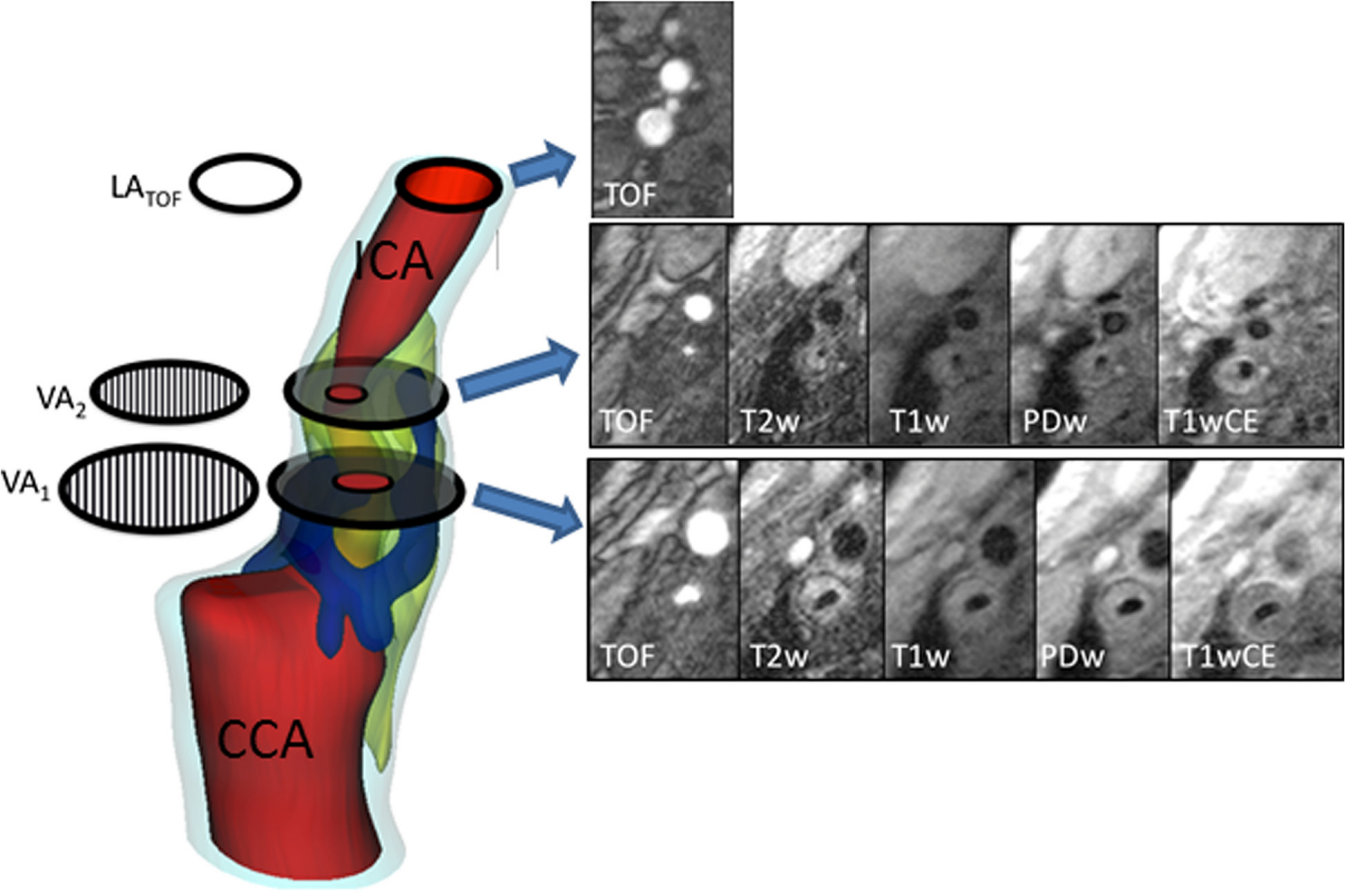
Illustration shows plaque with stenosis and expansive remodeling; the modified remodeling index (mRI) was calculated by dividing the outer vessel area at the proximal internal carotid artery VA_1_ (vessel-area) and VA_2_ by the lumen-area (LA) measured on TOF-images of a not affected and distally located part of the internal carotid artery (segment VA_1_ has higher mRI than VA_2_). The imaging protocol consisted of TOF and Black-Blood T2-, T1-, PD-images, and as contrast-enhanced (CE) T1w

The reviewer was blinded in regards to patients IDs and clinical information. Atherosclerotic plaques in the carotid arteries (on both the ipsilateral and the contralateral side of the stroke) were recorded and classified according to the modified criteria of the AHA. For definition of a complicated AHA Lesion-Type (LT) VI plaque, at least 1 of the following 3 criteria was required: fibrous cap rupture, intraplaque hemorrhage, or juxtaluminal hemorrhage/mural thrombus. Area measurements of the lumen, wall, vessel and tissue-components were obtained using a custom-designed semiautomatic image analysis tool CASCADE (University of Washington, Seattle, Washington). The normalized wall index (NWI) was calculated by dividing the wall area by the total vessel area. Tissue-components (lipid-rich NC, calcification, and hemorrhage) and presence/absence of ulcer were identified on the basis of previously published criteria [13, 14]. The modified remodeling index (mRI) was calculated by dividing the average outer vessel area of the internal carotid artery by the lumen area measured on TOF-images in a reference segment at the distal internal carotid artery of the same side (Fig. 1).

### Artery classification and analysis

Arteries were classified according to their most relevant AHA-LT {order of vulnerability: 1) AHA-LT VI, 2) AHA-LT IV/V, 3) AHA-LT VII, 4) AHA-LT III, 5) AHA-LT I}. The symptomatic artery was assigned to the side of ipsilateral DWI lesions. Artery analysis was performed for the ICA in a slice-based (correlation of mRI regarding vessel-dimensions and plaque-morphology; differences of mRI and vessel-dimensions regarding AHA-LT) and in an artery-based fashion (comparison of mean values of the respective vessel in symptomatic vs. asymptomatic arteries or stenotic vs. non-stenotic arteries).

### Statistical analysis

Categorical variables are presented as absolute and relative frequencies; continuous variables are presented as mean ± SD. A student’s t-test and one-way analysis of variance (ANOVA) were used to determine a mean difference of continues variables between groups. Multiple comparisons were Bonferroni-adjusted . Categorical data was analysed with Fisher’s exact test for comparison of proportions. Predictors of the modified remodeling index of the ICA were assessed using multiple linear regression analysis with cardiovascular risk factors (hypertension, smoking, hypercholesterolemia). Correlations were measured with Pearson’s r. A *p*-value of <0.05 (two sided) was chosen to indicate the level statistical significance. Statistical analysis was performed using SPSS for Windows, Version19.0 (IBM, Chicago/IL, USA).

## Results

### Patient characteristics

One hundred sixteen patients fulfilled the inclusion criteria. Five patients were excluded because of incomplete or insufficient CMR-data, which resulted in an inclusion of 111 patients with acute ischemic stroke (inclusion-rate = 94 %). Table 1 gives an overview of demographics and cardiovascular risk factors of the study population. 20 ICA could not be analyzed due to occlusion (*n* = 5), poor image-quality (*n* = 3), incomplete coverage (*n* = 2), and due to large angle (>65°) of the bifurcation (*n* = 10). AHA-LT III was detected in 75 % (86/111) of all patients, AHA-LT IV/V and VII had a prevalence of 46 % (51/111) and 41 % (45/111), respectively. The high-risk AHA-LT VI, defined by the features of luminal surface-defect, intraplaque hemorrhage or thrombus was noticed in 21 % (23/111) of the patients. Significant luminal stenosis (>50 %) was present in 33 % (37/111) of all patients. Regression analysis showed that regarding mRI there existed no relevant clinical cofactors like hypertension, smoking, and hypercholesterolemia.

**Table 1:** Patient demographics in detail

Patient characteristics	%	n	Mean ± SD	Range
Age			71.8 ± 10.3	38–94
Male	66.7	74/111		
Body Mass Index			25.8 ± 4.4	16.9–41.3
Arterial hypertension	71.2	79/111		
Current smoker	21.6	24/111		
Past smoker	36.0	40/111		
Coronary heart disease	37.8	42/111		
Hypercholesterolemia	41.4	46/111		
Diabetes mellitus	17.1	19/111		
Stenosis > 50 %	33.3	37/111		

### AHA-LT based analysis of the mRI

**Fig. 2:**
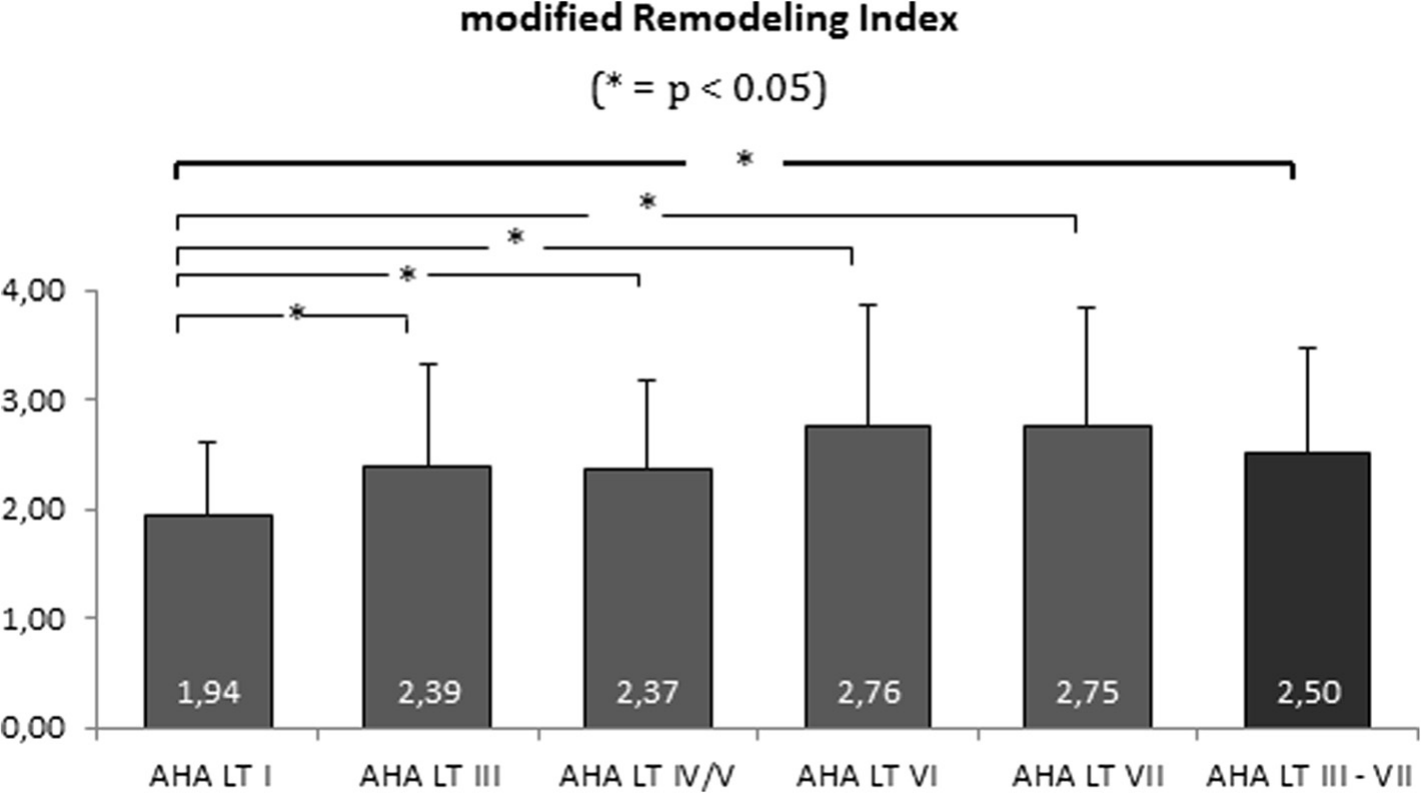
The modified remodeling-index was significantly higher in atherosclerotic arterial segments than in normal arterial segments (AHA-LT I). There was no significant difference between the AHA LT III – VII

**Fig. 3:**
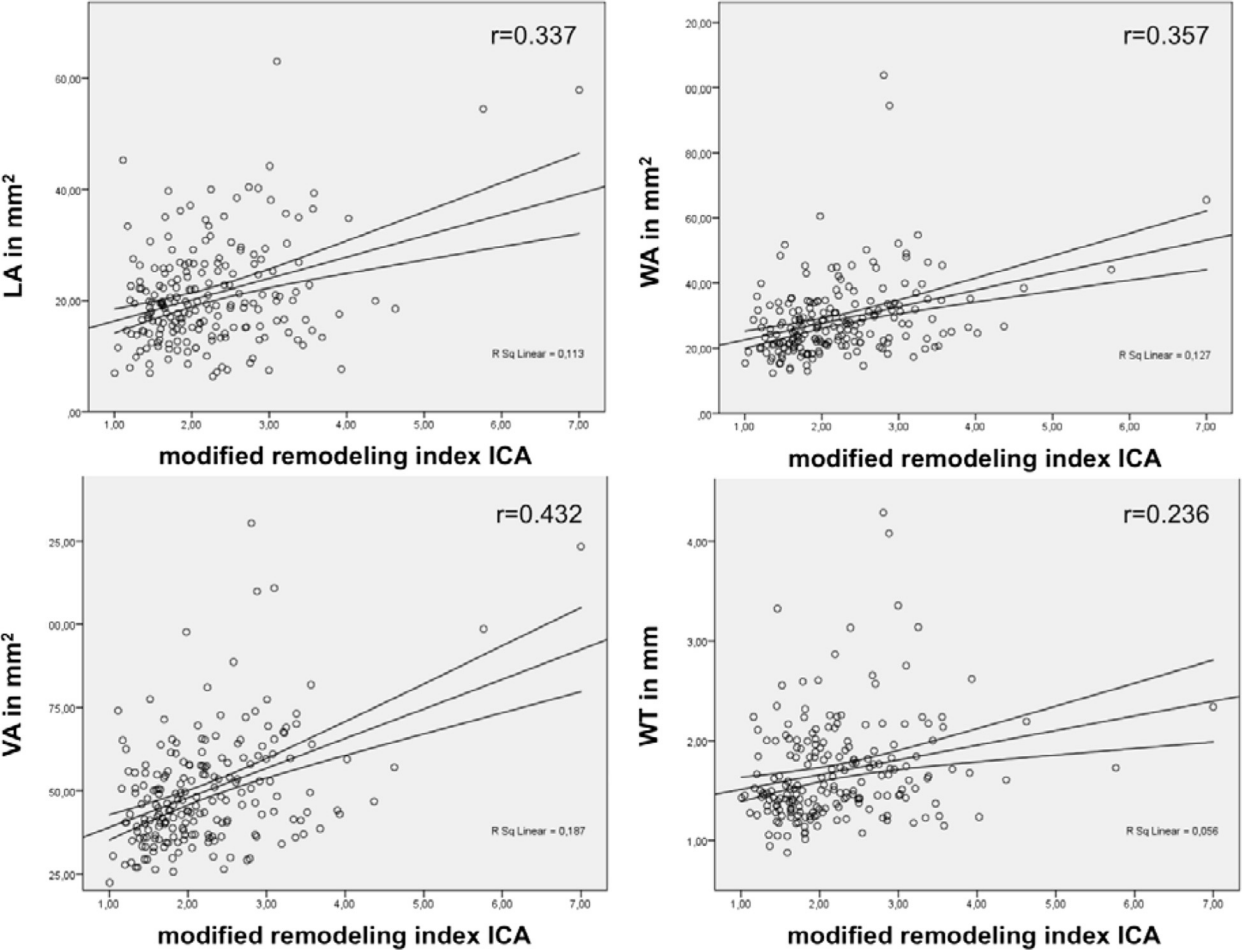
Scatterplots with significant correlations of modified remodeling index of the ICA with lumen area (LA), wallarea (WA), vesselarea (VA) and wallthickness (WT); *p*-value <0.001

ANOVA showed that all AHA-LT were significantly different in regards to CMR based quantitative plaque measurements (i.e. lumen area, wall area, vessel area, NWI, wall thickness and mRI ; *p* < 0.0001, Table 2). The mRI of the ICA in particular cases (Bonferroni correction) was significantly higher in atherosclerotic artery segments (defined as AHA-LT III or higher, mRI = 2.50 SD 0.97; range 1.02–7.00) when compared to normal artery segments, classified as AHA-LT I (mRI = 1.94 SD 0.67; range 0.87–4.49; *p* < 0.0001; Fig. 2). However, there was no significant difference of mRI, when comparing AHA-LT III, IV/V, VI and VII (mRI = 2.39 vs. 2.37 vs. 2.76 vs. 2.75; Fig. 2). NWI and wall thickness were the CMR based plaque parameters, which showed the highest variance between the different groups of AHA-LT (NWI: ANOVA: F-value 77; R^2^ = 0.5; WT: ANOVA F-value 109; R^2^ = 0.6). Table 2 shows a summary of quantitative CMR measurements in different AHA-LT. Significant positive correlations of the mRI were found with wall- and vessel area (*r* = 0.36 and 0.43; *p* < 0.0005), lipid-rich necrotic core area (*r* = 0.23; *p* = 0.018), ulcer-area (*r* = 0.26; *p* = 0.0055) and wall thickness (*r* = 0.24; *p* = 0.0036), and lumen-area (*r* = 0.34, *p* = 0.0063). The positive correlations with respect to vessel dimensions are shown as scatter plots in Fig. 3.

**Table 2:** Summary of modified remodeling index (mRI) and vessel dimension measurements in different AHA-lesion-type

Patients (%; x/n)	AHA I 92.8 % (103/111)	AHA III 77.5 % (86/111)	AHA IV/V 45.9 % (51/111)	AHA VI 20.7 % (23/111)	AHA VII 40.5 % (45/111)	*p*-value	F-value	R^2^	Bonferroni correction (*p* < 0.05)
Modiefied remodeling index	1.94 ± 0.67	2.4 ± 0.93	2.37 ± 0.82	2.76 ± 1.10	2.75 ± 1.09	<0.0001	9.21	0.11	AHA LTs: I vs III; I vs IV/V; I vs VI; I vs VII
Lumen area [mm^2^]	20.8 ± 8.7	22.4 ± 0.5	15.1 ± 7.8	15.4 ± 7.1	21.4 ± 10.7	<0.0001	6.74	0.08	AHA LTs: I vs IV/V; III vs IV/V; III vs VI; IV/V vs VII
Wall area [mm^2^]	21.4 ± 4.9	31.1 ± 8.0	39.5 ± 13.0	52.8 ± 34.5	34.8 ± 10.0	<0.0001	39.85	0.34	AHA LTs: I vs III; I vs IV/V; I vs VI; vs VII; III vs IV/V; III vs VI; IV/V vs VI; VI vs VII
Outer wall area [mm^2^]	42.3 ± 13.1	53.5 ± 18	54.6 ± 17.0	68.3 ± 38.9	56.2 ± 18.4	<0.0001	12.16	0.14	AHA LTs: I vs III; I vs IV/V; I vs VI; I vs VII; III vs VI; IV/V vs VI
Normalized wall index	0.53 ± 0.06	0.6 ± 0.08	0.73 ± 0.09	0.76 ± 0.08	0.63 ± 0.10	<0.0001	76.83	0.50	AHA LTs: I vs III; I vs IV/V; I vs VI; I vs VII; III vs IV/V; III vs VI; IV/V vs VII; VI vs VII
Wall thickness [mm]	1.3 ± 0.1	1.8 ± 0.2	2.4 ± 0.6	2.8 ± 0.9	2.0 ± 0.4	<0.0001	108.80	0.59	AHA LTs (all): I vs III; I vs IV/V; I vs VI; I vs VII; III vs IV/V; III vs VI; III vs VII; IV/V vs VI; IV/V vs VII; VI vs VII

### Analysis of the mRI in symptomatic and asymptomatic arteries

**Fig. 4:**
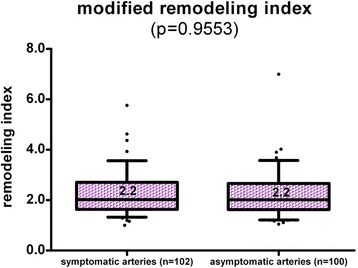
No difference of the mRI was observed in symptomatic and asymptomatic arteries

**Fig. 5:**
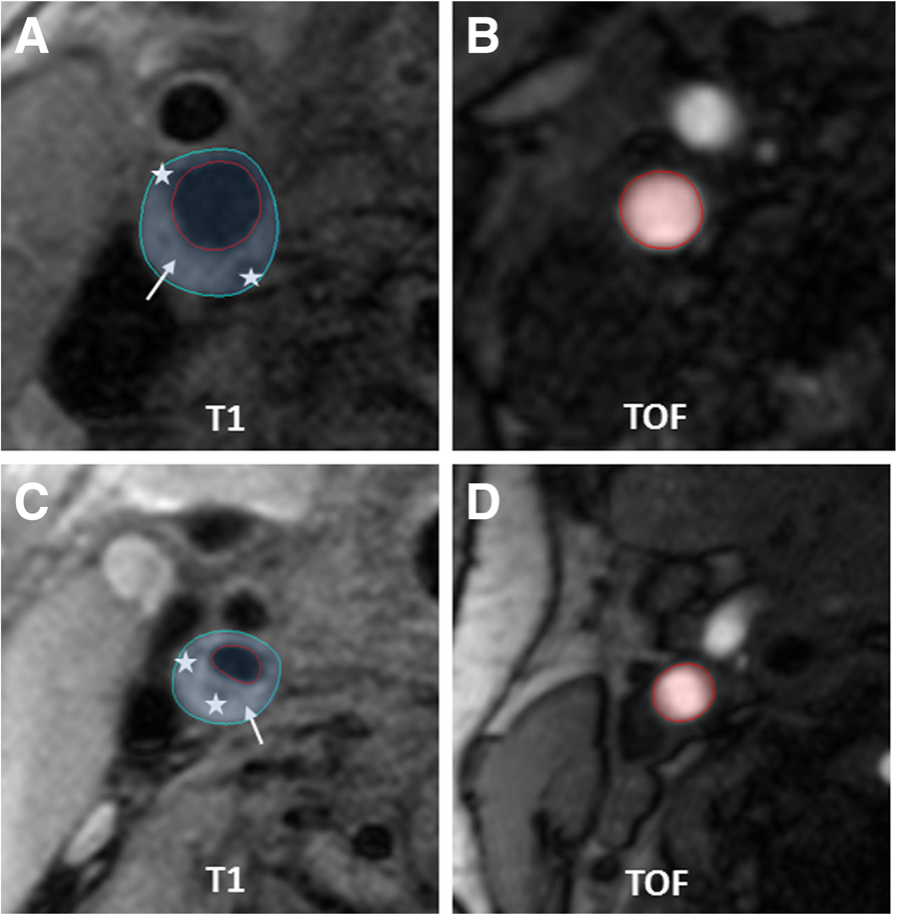
Asymptomatic (**a**, **b**) and symptomatic (**c**, **d**) internal carotid arteries (ICA) with AHA-LT V (white arrow = lipid-rich NC, white star = and small calcifications). Modified remodeling index (mRI) was calculated by dividing the outer vessel area in the T1-image (area within the blue circle, A:67.2 mm^2^; C:46.7 mm^2^) with the lumen-area in the TOF-angiography 40 mm distal to bifurcation in a normal configurated vessel segment (area within the red circle, B:24.4 mm^2^,D:25.7 mm^2^). Relatively high mRI (2.77) in asymptomatic (**a**, **b**) and relatively low mRI (1.81) in symptomatic artery

A statistical comparison of symptomatic arteries (ipsilateral to DWI lesion) and asymptomatic arteries (contralateral to DWI lesion) revealed no difference of mRI (symptomatic and asymptomatic side: 2.2, *p* = 0.955; Fig. 4). Fig. 5 shows a symptomatic plaque with small mRI as well as a asymptomatic plaque with high mRI, respectively.

There were few significant differences in AHA-LT prevalence and MR-plaque measurements. AHA-LT VI plaques were more often seen in symptomatic arteries (20 vs. 4 %, *p* = 0.0008). All other AHA-LT’s showed no significant different prevalence in symptomatic compared to asymptomatic arteries. In terms of assessment of plaque-components, intraplaque hemorrhage (19 vs 4 %, *p* = 0.0014) and lipid-rich NC (39 vs. 22 % *p* = 0.0095) were also significantly more common in symptomatic arteries compared to asymptomatic arteries. Calcified plaques showed nearly similar frequency in both groups (30 vs. 34 %, *p* = 0.652). Symptomatic arteries had a higher NWI (0.60 vs. 0.56 *p* = 0.005) and a thicker vessel wall (1.8 vs. 1.6 mm *p* = 0.022). Lumen area, wall area, vessel area and the mRI of the ICA were not significantly different between symptomatic and asymptomatic arteries. The statistical comparison of the symptomatic and asymptomatic artery is shown in detail in Table 3.

**Table 3:** Analysis of the modified remodeling index (mRI), vessel dimensions, AHA-lesion-type distribution, and plaque-components in symptomatic and asymptomatic arteries

Vessel dimensions (mean)	Symptomatic ICA (*n* = 102)	Asymptomatic ICA (*n* = 100)	*p*-value
Modified remodeling index	2.22 ± 0.80	2.21 ± 0.85	n.s.
Lumen area [mm^2^]	19.8 ± 9.4	22.1 ± 9.2	n.s.
Wall area [mm^2^]	29.9 ± 13.4	27.7 ± 9.7	n.s.
Outher wall area [mm^2^]	49.7 ± 18.0	49.8 ± 15.9	n.s.
Normalized wall index	0.60 ± 0.11	0.56 ± 0.09	0.0056
Wall thickness [mm]	1.8 ± 0.6	1.6 ± 0.4	0.0221
AHA lesion type distribution			
AHA LT I (%; x/n)	23.5 % (24/102)	25.0 % (25/100)	n.s.
AHA LT III (%; x/n)	19.6 % (20/102)	27.9 % (27/100)	n.s.
AHA LT IV/V (%; x/n)	20.6 % (21/102)	18.0 % (18/100)	n.s.
AHA LT VI (%; x/n)	19.6 % (20/102)	4.0 % (4/100)	0.0008
AHA LT VII (%; x/n)	16.7 % (17/102)	26.0 % (26/100)	n.s.
Plaque components			
Intraplaque hemorrhage (%; x/n)	18.6 % (19/102)	4.0 % (4/100)	0.0014
Lipidrich necrotic core (%; x/n)	39.2 % (40/102)	22.0 % (22/100)	0.0095
Calcification (%; x/n)	30.4 % (31/102)	34.0 % (34/100)	n.s.
Ulceration (%; x/n)	5.9 % (6/102)	1.0 % (1/100)	n.s.
Thrombus (%; x/n)	2.0 % (2/102)	0.0 % (0/100)	n.s.

### Analysis of the mRI in arteries with and without significant luminal stenosis

There was no significant difference of the mRI of the ICA between stenotic and non-stenotic arteries (stenotic artery mRI 2.22 vs. non-stenotic artery mRI 2.21; *p* = 0.9383, Fig. 5) and there was no significant difference between stenotic symptomatic arteries (*n* = 28, mRI 2.1 SD 0.8) and stenotic asymptomatic arteries (*n* = 16, mRI 2.4, SD 0.8; *p* = 0.434, Fig. 4). Student’s t-test between stenotic (luminal stenosis ≥50 %, 44/202) and non-stenotic (158/202) arteries revealed smaller lumen-area (13.7 vs. 23 mm^2^*p* < 0.0001), an increased NWI (0.69 vs. 0.55 *p* < 0.0001) and an increased wall thickness (2.1 vs. 1.6 mm *p* = 0.0016). Stenotic arteries had a higher prevalence of complicated AHA-LT VI (25 vs. 8 %; *p* = 0.006) and AHA-LT IV/V (39 vs. 14 %; *p* < 0.0001). Consequently, stenotic arteries also had a higher frequency of intraplaque hemorrhage (25 vs. 8 %; *p* = 0.0029) and lipid-rich NC (64 vs. 22 %; *p* < 0.0001). The statistical comparison of arteries with and without significant stenosis is shown in detail in Table 4.

**Table 4:** Analysis of the modified remodeling index, vessel dimensions, AHA-LT distribution, and plaque-components of internal carotid arteries (ICA) with and without significant luminal stenosis (>50 %)

Vessel dimensions mean	Stenotic ICA (>50 %) (*n* = 44)	Non-stenotic ICA (*n* = 158)	*p*-value
Modifed remodeling index	2.22 ± 0.82	2.21 ± 0.83	n.s.
Lumen area [mm^2^]	13.7 ± 5.50	23.0 ± 9.2	<0.0001
Wall area [mm^2^]	32.1 ± 14.0	27.9 ± 10.9	0.0371
Outher wall area [mm^2^]	45.8 ± 16.3	50.9 ± 17.1	n.s.
Normalized wall index	0.69 ± 0.09	0.55 ± 0.08	<0.0001
Wall thickness [mm]	2.1 ± 0.60	1.6 ± 0.4	<0.0001
AHA lesion type distribution			
AHA LT I (%; x/n)	0.0 % (0/44)	29.8 % (47/158)	<0.0001
AHA LT III (%; x/n)	18.2 % (8/44)	26.0 % (41/158)	n.s.
AHA LT IV/V (%; x/n)	38.6 % (17/44)	13.9 % (22/158)	0.001
AHA LT VI (%; x/n)	25.0 % (11/44)	8.2 % (13/158)	0.006
AHA LT VII (%; x/n)	18.2 % (8/44)	22.2 % (35/138)	n.s.
Plaque components			
Intraplaque hemorrhage (%; x/n)	25.0 % (11/44)	7.6 % (12/158)	0.0029
Lipidrich necrotic core (%; x/n)	63.6 % (28/44)	21.5 % (34/158)	<0.0001
Calcification (%; x/n)	50.0 % (22/44)	27.2 % (43/158)	0.0060
Ulceration (%; x/n)	9.1 % (4/44)	1.9 % (3/158)	0.0417
Thrombus (%; x/n)	2.3 % (1/44)	0.6 % (1/158)	n.s.

### Slice based Analysis of the mRI at location of maximal luminal stenosis

202 single slices were selected in regards to location of maximal luminal stenosis (one slice per artery). Values and standard deviation for mean mRI, lumen area, wall area, total vessel area were 2.02 ± 0.76, 15.7 ± 8.7, 30.8 ± 14.4 and 46.5 ± 18.5, respectively. This slice based mRI showed moderate correlations with total vessel area, wall area and wall thickness (*r* = 0.50, 0.49, and 0.40 respectively; *p* < 0.0001), weak correlations with lumen area and NWI (*r* = 0.25 and 0.17; *p* < 0.05). Different AHA lesion types had significant different mRI measurements (AHA LT I: 1.72, AHA LT III: 2.03, AHA LT IV/V: 2.09, AHA LT VI: 2.46, AHA LT VII: 1.87; ANOVA: F-value 5.4; R2 = 2.9; *p* < 0.0001). Post hoc testing revealed specific differences between AHA LT I vs. VI and AHA LT VI vs VII (bonferroni corrected, *p* < 0.05). There was no significant difference between symptomatic and asymptomatic arteries (mRI 2.08 vs 1.97, *p* = 0.31), as well as between arteries with >50 % luminal stenosis and arteries <50 % luminal stenosis (mRI 2.03 vs 2.02, *p* = 0.92).

## Discussion

In this prospective, monocentric observational study expansive remodeling of the carotid arteries was correlated with plaque morphology, grade of stenosis and symptom status using in-vivo MR-imaging. Our results showed that expansive arterial remodeling does exist in the carotid arteries and that carotid arteries with atherosclerotic plaques have a significant higher mRI compared to non-diseased vessel segments. Significant differences with regard to vessel dimensions, prevalence of distinct AHA-LT, and plaque-components were observed between symptomatic vs. asymptomatic arteries and stenotic vs. non-stenotic arteries, respectively. However, there was no significant difference of the mRI with regard to clinical symptoms, stenosis and AHA-LT with atherosclerosis. The slice-based analysis (location with maximal luminal stenosis) of the mRI thereby showed similar findings to the artery based analysis.

The mean mRI in our study was 1.9 for non-affected and 2.5 for affected vessel-segments. This basically is in concordance with a CTA based study of Kamenskiy et al., who investigated the age- and disease-related geometry of the carotid arteries. Based on their measurements (outer vessel diameter of the carotid bulb and outer diameter of the distal ICA) the remodeling index as well was lower for non-diseased versus diseased ICA’s (1.7 versus 2.2). A high variability of the diameter of the carotid bulb was found for both the healthy and the diseased individuals (5–11 versus 6.5–12 mm) [15]. Though, these interindividual variations in the geometry of the carotid bifurcation seem to increase with aging as well as atherosclerotic disease progression [16].

Studies of the coronary arteries have shown a positive correlation of expansive remodeling with unstable coronary syndromes suggesting that expansive remodeling is associated with higher plaque-vulnerability [2–5]. Consecutively, in a consensus document by a group of experienced researchers in atherosclerosis (pathologists, clinicians, molecular biologists, imaging scientists) based on studies of the coronary arteries beside plaque-features like e.g. intraplaque hemorrhage expansive remodeling as well was described as a minor criterion for the vulnerable plaque [17, 18].

In contrast to previous studies of the coronary arteries we did neither observe a correlation of carotid expansive remodeling with morphological features of plaque-vulnerability nor with clinical symptoms. A possible explanation for this discrepancy could be the fact that plaque-morphology as well as the progression of atherosclerosis generally differs within the respective vascular bed. Dalager et al. e.g. performed an autopsy study addressing different vascular beds (carotid arteries, femoral arteries, coronary arteries) and could show that there existed distinct pattern of atherosclerosis in the respective arteries. In the coronary arteries e.g. the affected intima was diffusely thickened compared to the intima in other vascular beds [9]. This alteration might represent a weakening of the intima and therefore could be the reason for a higher risk of plaque rupture in the coronary circulation, especially in case of expansive remodeling and plaques with large lipid-rich NC. Given the intact intima, the lumen preserving effect of expansive remodeling in the carotid arteries contrarily might outbalance the higher risk of rupture.

Another explanation could be the different hemodynamic of the two regions, since in the region of the carotid bulb there usually occurs relatively low wall shear stress with unidirectional and axially not aligned flow patterns that oscillates between −7 and +4 dynes/cm^2^ [19, 20].

Therefore, even large plaques with expansive remodeling might be less prone to rupture compared to the coronary arteries with higher wall-shear stress with up to 44 dynes/cm^2^ [21, 22].

Regarding the type of remodeling there as well seems to be a substantial difference between the distinct vascular beds. Prospective in-vivo CMR studies of progression of atherosclerosis have shown that the carotid arteries showed a constrictive remodeling pattern, whereas the superficial femoral artery and abdominal aorta tend to compensate an increasing plaque-burden by significantly higher grade of expansive remodeling [23, 24]. Thus, expansive remodeling generally could be less frequent in the carotid arteries than in other locations explaining the lower clinical impact compared to previous studies of the coronary arteries.

In contrast to our results recent in-vivo studies showed a significant correlation of expansive remodeling with clinical symptoms, indicating that expansive remodeling is associated with plaque-vulnerability [25–27]. However, patients included in the aforementioned studies were sent for carotid endarterectomy and/or showed significant stenosis of the carotid artery (average grade of stenosis >70 %). Thus, the included patients had a considerable higher grade of atherosclerosis compared to our study. The increased expansive remodeling in case of more advanced atherosclerosis might e.g. more often be the result of acute hemorrhage into a plaque, which is more frequently seen in symptomatic compared to asymptomatic patients [28]. Thus, increased expansive remodeling in these cases would rather be a marker for previously ruptured plaques than a good predictive criterion for clinical symptoms. Further possible reasons for the absence of correlation of mRI with clinical symptom status might be that carotid arteries with advanced atherosclerotic disease have a higher tendency for inward remodeling compared to the coronary arteries. This would explain that in our study outer wall area in arteries with >50 % stenosis was lower than outer wall area in arteries with <50 % stenosis. Finally, due to the specific and unique anatomical situation in the carotid bulb remodeling in the carotid arteries might be much less important in the carotid arteries compared to the coronary arteries. Two of the in-vivo studies which showed correlation of expansive remodeling and clinical symptoms [25, 26] are based on CTA-data, which is not considered to be the standard of reference for quantitative evaluation of the vessel wall. This as well reduces the comparability with the results of our study. Our data on the contrary is supported by two CMR-studies of symptomatic patients with significant carotid artery atherosclerosis which revealed no significant difference in mean outer wall area (which is an indirect parameter for expansive remodeling) between symptomatic and asymptomatic carotid arteries [27, 29].

New data regarding coronary lesions as well poses the question on the common approach of separating plaques into two groups with positive and negative remodeling, respectively. In the PROSPECT study of 697 consecutive patients with acute coronary syndrome it has been shown that both, negative and positive remodeling was connected with a higher rate of major cardiac clinical events [30].

This study has several potential limitations. The mRI was calculated by dividing the outer vessel area at the internal carotid artery plaque by the lumen area measured on TOF-images (vessel wall not visible) in a distally located normal appearing reference segment of the same artery. Therefore, it was assumed that the distal part of the internal carotid artery neither shows a change of the lumen nor exhibits atherosclerotic plaques. Furthermore, unlike in the coronaries, the internal carotid artery partially consists of the carotid bulb, which shows a physiological widening. However, the natural variability of the caliber of the carotid bulb to the distal part of the internal carotid artery should be equalized between the symptomatic and asymptomatic site.

## Conclusion

Expansive arterial remodeling is present in atherosclerotic plaques of the internal carotid artery. However, there was no significant association with regard to symptomatic arteries, AHA-LT or presence of significant luminal stenosis. Hence, results of our study suggest that expansive arterial remodeling is not a very practical marker for plaque-vulnerability in the carotid arteries.

## Abbreviations

AHA: American Heart Association
AHA-LT: AHA-Lesion-Type
ANOVA: Analysis of variance
CMR: Cardiovascular Magnetic Resonance
CT: Computed tomography
DWI: Diffusion-weighted-imaging
ICA: Internal carotid artery
LE: Lesion type
MR: Magnetic resonance
mRI: Modified remodeling index
MRI: Magnetic resonance imaging
NC: Necrotic core
NWI: Normalized wall index
TOF: Time-of-flight
